# Optical Dielectrophoretic (DEP) Manipulation of Oil-Immersed Aqueous Droplets on a Plasmonic-Enhanced Photoconductive Surface

**DOI:** 10.3390/mi13010112

**Published:** 2022-01-11

**Authors:** Si Kuan Thio, Sung-Yong Park

**Affiliations:** 1 Department of Mechanical Engineering, National University of Singapore, Singapore 117575, Singapore; sikuan@u.nus.edu; 2 Department of Mechanical Engineering, San Diego State University, San Diego, CA 92182-1323, USA

**Keywords:** dielectrophoresis (DEP), optoelectronic tweezers (OET), droplets, TiOPc

## Abstract

We present a plasmonic-enhanced dielectrophoretic (DEP) phenomenon to improve optical DEP performance of a floating electrode optoelectronic tweezers (FEOET) device, where aqueous droplets can be effectively manipulated on a light-patterned photoconductive surface immersed in an oil medium. To offer device simplicity and cost-effectiveness, recent studies have utilized a polymer-based photoconductive material such as titanium oxide phthalocyanine (TiOPc). However, the TiOPc has much poorer photoconductivity than that of semiconductors like amorphous silicon (a-Si), significantly limiting optical DEP applications. The study herein focuses on the FEOET device for which optical DEP performance can be greatly enhanced by utilizing plasmonic nanoparticles as light scattering elements to improve light absorption of the low-quality TiOPc. Numerical simulation studies of both plasmonic light scattering and electric field enhancement were conducted to verify wide-angle scattering light rays and an approximately twofold increase in electric field gradient with the presence of nanoparticles. Similarly, a spectrophotometric study conducted on the absorption spectrum of the TiOPc has shown light absorption improvement (nearly twofold) of the TiOPc layer. Additionally, droplet dynamics study experimentally demonstrated a light-actuated droplet speed of 1.90 mm/s, a more than 11-fold improvement due to plasmonic light scattering. This plasmonic-enhanced FEOET technology can considerably improve optical DEP capability even with poor-quality photoconductive materials, thus providing low-cost, easy-fabrication solutions for various droplet-based microfluidic applications.

## 1. Introduction

Microfluidic systems have drawn great interest in numerous chemical and biological applications due to their capability to precisely manipulate micro/nanoscopic particles, including cells, droplets, and colloids [1,2,3]. Particle manipulation in microfluidic devices represents an essential step for subsequent processes such as concentration, separation, sorting, and detection of samples [4,5,6]. To flexibly control the particle’s movement, various active-type technologies have been explored, including optical tweezers [7,8], dielectrophoresis (DEP) [9,10,11], electrowetting-on-dielectric (EWOD) [12,13,14], magnetic tweezers [15,16], and acoustic traps [17,18]. Among these technologies, DEP stands out as a low-cost, label-free approach widely used for particle manipulation without surface contact, thus minimizing potential contamination issues [19,20,21]. DEP refers to a phenomenon where a force is exerted on a dielectric particle interacted with spatial gradient (i.e., non-uniformity) of the electric field. The strength of the DEP force is determined by various parameters such as the particle’s shape and size, the electrical properties of the particle and the suspending medium, and the frequency of the electric field [19]. Consequently, these control parameters provide DEP manipulation with high flexibility and selectivity, even control of the particle’s orientation [22]. However, DEP-driven microfluidic devices often encounter the issues of complex wiring and interconnection of electrodes to accomplish two-dimensional (2D) parallel manipulation [20,23]. This significantly complicates the fabrication process, increases the cost for disposable chips, and limits DEP applications in many chemical and biological analyses.

In recent years, an optical DEP method called optoelectronic tweezers (OET) has been developed as a low-cost alternative to address 2D wiring issues in microfluidic systems [24,25,26]. Chiou et al. have first demonstrated the light-driven DEP concept for parallel manipulation of microscopic particles [24]. Upon the illumination of light patterns onto a photoconductive surface, incoming light rays were absorbed to increase their photo-state conductivity. As a consequence, a non-uniformity of the electric field was created for DEP operation due to the locally modified electric impedance. They have experimentally demonstrated the generation of optical traps for large-scale parallel manipulation of 15,000 particles on a 1.3 × 1.0 mm^2^ area without any complex wiring and interconnection issues. OET technologies have been further advanced for single-cell manipulation suspended in high-conductivity physiological buffers such as cell culture media [25] as well as large-scale assembling of metallic nanowires [26]. Due to an impedance matching issue, however, conventional OET devices were not able to control the electric field in the layer of an electrically insulating oil medium that was typically used in droplet-based microfluidic platforms [27,28,29]. To address this drawback, Park et al. developed a floating electrode optoelectronic tweezers (FEOET) mechanism [30]. By addressing a light pattern onto a coplanar photoconductive surface, a laterally applied electric field was locally modified to create a positive dielectrophoretic force for optical droplet manipulations, including biological or biochemical samples surrounded by an oil medium. Using a FEOET principle, numerous droplet-based microfluidic functions (e.g., droplet transporting, mixing, merging, parallel processing, and integration with microwell structures) have demonstrated the benefits of device simplicity and functionality [31]. 

One key requirement for previous optical DEP devices [24,26,30,31,32] was the use of a high-quality photoconductive material like amorphous silicon (a-Si) to perform light-driven DEP operation. However, the fabrication of a thin-film a-Si layer typically requires high-cost and complex facilities such as chemical vapor deposition (CVD) or plasma enhanced chemical vapor deposition (PECVD), leading to device complexity and cost ineffectiveness [33]. To address the fabrication issues, recent studies have utilized a polymer-based photoconductive material such as titanium oxide phthalocyanine (TiOPc) [34,35,36,37]. With the polymer-based material, its thin-film layer can be easily fabricated via an inexpensive, spin-coating fabrication step without the need for high-cost and complex facilities. A crucial drawback of the TiOPc for optical DEP applications, however, is its low-quality photoconductivity due to the poor light absorption performance. Consequently, the photoconductive performance of the TiOPc is a few orders poorer than that of the a-Si [37,38,39,40].

In this study, we present a plasmonic field enhancement to greatly improve light absorption performance of the low-quality TiOPc material and thus result in an enhanced dielectrophoretic force for effective manipulation of an oil-immersed aqueous droplet on a FEOET device. With the use of plasmonic nanoparticles as light scattering elements above the TiOPc layer, light rays transmitted through the TiOPc experience scattering over a wide angular spread and effectively increase their optical path lengths. They were then diverted back onto the TiOPc layer to significantly enlarge its light absorption and photoconductivity. As a result, the FEOET device can improve optical DEP performance for effective manipulation of an oil-immersed aqueous droplet on a light-patterned photoconductive surface with an enhanced dielectrophoretic force. Two numerical simulation works have been conducted to understand a plasmonic-enhanced DEP phenomenon on a FEOET device. Optics simulation showed how incident rays are scattered from plasmonic nanoparticles over an angular spread to increase their optical path lengths. Further simulations on electric field distribution supported the non-uniform field enhancement necessary for DEP manipulation. A spectrophotometric measurement study has verified largely improved light absorption onto a TiOPc layer. An additional experimental study showed a light-actuated droplet’s movement at a speed of as fast as 1.90 mm/s (a more than 11-fold improvement) due to the plasmonic field enhancement effect. Using plasmonic nanoparticles as light scattering elements can considerably enlarge optical DEP performance even with low-quality photoconductive materials, thus offering low-cost and simple FEOET devices for a broad range of droplet-based microfluidic applications.

## 2. Device Fabrication and Its Working Principle

**Figure 1:**
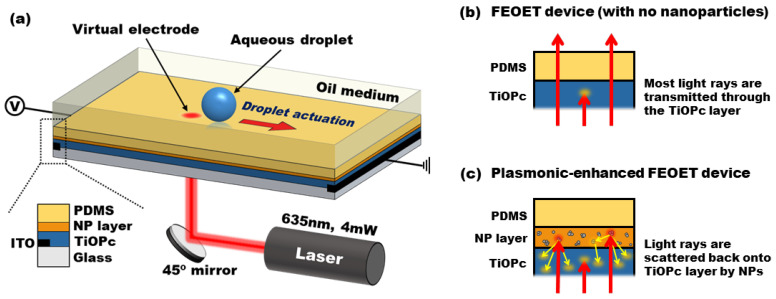
**A schematic of the plasmonic-enhanced FEOET device.** (**a**) The device consists of an 8-µm TiOPc layer on top of which metallic nanoparticles are dispersed. The thickness of a nanoparticle (NP) layer is varied to understand the thickness effect on plasmonic-enhanced DEP performance of the device. An open PDMS chamber with its thickness of 12.5 µm is finally placed above the NP layer to house droplets surrounded by an oil medium. (**b**) For the FEOET device without the nanoparticle layer, only a small portion of the input optical rays can be absorbed on the TiOPc layer due to its poor photoconductive property. Most of the rays just pass through it before making a contribution to its photo-state conductivity, resulting in poor DEP performance. (**c**) However, by using plasmonic nanoparticles as light scattering elements above the TiOPc layer, the transmitted rays experience light scattering and effectively increase their optical path lengths over a wide angular spread. Then, more light rays are redirected and absorbed within the TiOPc layer, thus significantly enhancing the photoconductivity. As a consequence, the DEP performance of the FEOET device can be greatly improved for effective optical droplet manipulation.

Figure 1 shows a schematic illustration of the FEOET device that allows plasmonic-enhanced DEP manipulation of droplets suspended in an oil medium. It simply consists of an open stationary chamber filled with oil above several single-sided and featureless layers. Device fabrication begins with indium tin oxide (ITO) electrodes patterned at both sides of a glass substrate with a 30 mm gap. For the photoconductive layer, a titanium oxide phthalocyanine (TiOPc) solution was prepared by dissolving TiOPc powder (Sigma-Aldrich, St. Louis, MO, USA) in a chlorobenzene solvent (1.0 wt%) at 80 °C for a period of 2 h. This synthesized TiOPc solution was then drop-casted and left to dry at room temperature for another 2 h, after which a TiOPc layer with 8 µm in thickness was obtained above the ITO electrodes. With the TiOPc layer, light-driven DEP performance is very limited because of its much poorer photoconductive property, as compared to semiconductors like a-Si. To improve the photoconductive performance of the FEOET device even with the low-quality TiOPc layer, this study utilized metallic nanoparticles as light scattering elements on top of the TiOPc layer. Experimentally, aluminum (Al) nanoparticles sizing ∼50 nm in diameter were used to be dissolved in a 6% Teflon solution (AF 1601S, DuPont, Wilmington, DE, USA). To facilitate the dispersion of the nanoparticles, the solution was further diluted to 3% with Fluoroinert Liquid (FC-72, 3M, Saint Paul, MN, USA) and subsequently spun-coated on top of the TiOPc layer and cured at 110 °C for another 2 h. To understand the thickness effect of a nanoparticle layer on optical DEP performance, it was varied by controlling the spin speeds of a nanoparticle solution. Finally, an open polydimethylsiloxane (PDMS) chamber with a 12.5-µm thickness was placed above the nanoparticle layer to house an aqueous droplet surrounded by an oil medium.

A droplet actuation principle on a FEOET device is based on the electrostatic interaction of the aqueous droplet with the light-patterned photoconductive surface. This light-driven DEP actuation principle has been well described in the previous studies [30,31]. In brief, a direct current (DC) bias voltage is applied between two ITO electrodes positioned at both sides of the device. Subsequently, an electric field is uniformly generated along a lateral direction. In the absence of light illumination, this electric field remains uniform without any perturbation, where no DEP force can be produced. When a light beam is illuminated onto a photoconductive layer, the field induced electron-hole pairs locally modify its photo-state conductivity to function as a virtual electrode. Thus, the uniform field pattern originally formed can be strongly perturbed near the light illuminated area to create the non-uniform electric field for DEP manipulation. As an aqueous droplet is much more conductive than its surrounding oil medium, an electric dipole is induced by the charged ions accumulated on both edges of the droplet under the application of a lateral electric field. Although this field distribution is highly non-uniform, no DEP force can be induced because the field distribution is balanced around the droplet. When a light beam illuminates near one edge of the droplet (Figure 1a), a virtual electrode is locally created on the photoconductive layer and reduces the field strength at the illuminated area. This disrupts the originally balanced field distribution around the droplet and causes a positive DEP force to drive it away from the light illumination towards the direction of the stronger electric field region.

To further enhance optical DEP performance of the FEOET device, our approach is to utilize plasmonic nanoparticles as light scattering elements above the TiOPc layer. Without the nanoparticles (Figure 1b), most of the input rays just pass through the TiOPc layer before making a contribution to its photoconductivity due to the poor light absorption property of the TiOPc, resulting in weak DEP performance of the device. However, with the nanoparticle layer added (Figure 1c), the transmitted rays that have not been absorbed onto the TiOPc layer can undergo plasmonic light scattering over a high angular spread and effectively increase their optical path lengths in the layer [41,42]. Thus, more light rays can be redirected onto the TiOPc layer to greatly increase its light absorption and photoconductivity. This plasmonic effect enhances the field non-uniformity around the droplet, resulting in a greater DEP force for the effective manipulation of droplets.

## 3. Simulation Study

To qualitatively understand plasmonic-enhanced DEP performance of the FEOET device, we have conducted three-dimensional (3D) numerical simulation studies of plasmonic light scattering and electric field distribution using a finite-element software (COMSOL Multiphysics 5.5, Stockholm, Sweden). The first simulation study has focused on plasmonic light scattering by nanoparticles to increase the light absorption of the TiOPc layer and its photo-state conductivity. Another simulation study verifies the non-uniform field enhancement for DEP performance of FEOET devices. Comparative studies were also implemented for the simulations with and without the layer of plasmonic nanoparticles.

### 3.1. Plasmonic Light Scattering

**Figure 2:**
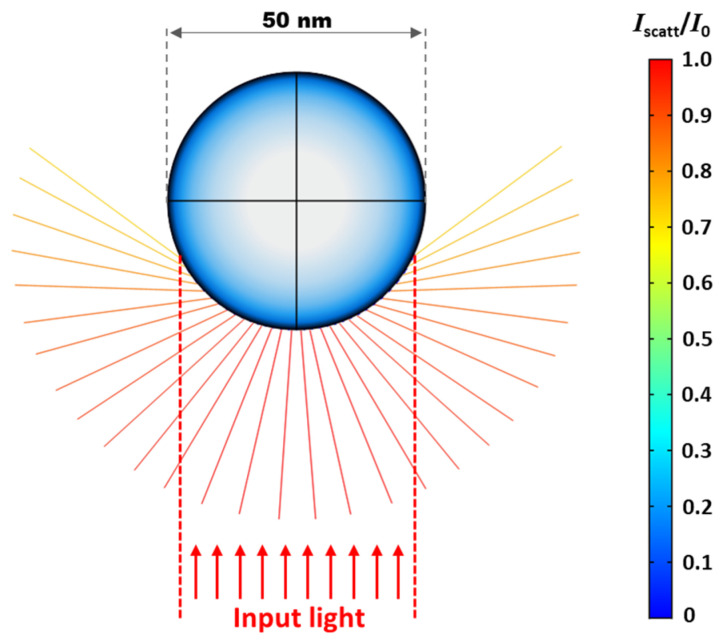
**Simulation results of plasmonic light scattering by a spherical nanoparticle with a diameter of 50 nm.** A total of 500 input rays are assumed to be illuminated from the bottom. Upon hitting the nanoparticle, these rays are scattered over a high angular spread, thereby increasing their optical path lengths. The intensity of each scattered ray is also observed to vary according to the direction of scatter. The larger the direction of scatter, the lower the scattered intensity of the ray (*I*_scatt_) as compared to the input ray (*I*_0_). A color scale bar indicates the intensity ratio between the scattered and input ray, i.e., *I*_scatt_/*I*_0_.

For the simulation study conducted on plasmonic light scattering, a spherical nanoparticle was first modelled to be electrically conductive with its diameter of 50 nm, which is comparable with the nanoparticles experimentally used. To represent the optical rays that were transmitted through without being absorbed onto the underneath photoconductive layer, a circular light beam with a total of 500 rays was assumed to be illuminated from below. Figure 2 shows a cross-sectional view of the light rays scattered by the nanoparticle over a wide angular spread to effectively increase their optical path lengths. It is worthwhile to note that the larger the direction of scatter, the lower the scattered intensity (*I*_scatt_) of the ray is observed as compared to the input ray (*I*_0_). The scattered intensity of each ray obtained in this simulation study does well match with the Rayleigh theory, describing that the angular dependence of plasmonic light scattering can be attributed to the nanoparticle’s absorption [43,44]. This simulation study demonstrates how input rays are angularly scattered by the nanoparticle and redirected back onto the photoconductive layer beneath to contribute to its light absorption and photo-state conductivity.

**Figure 3:**
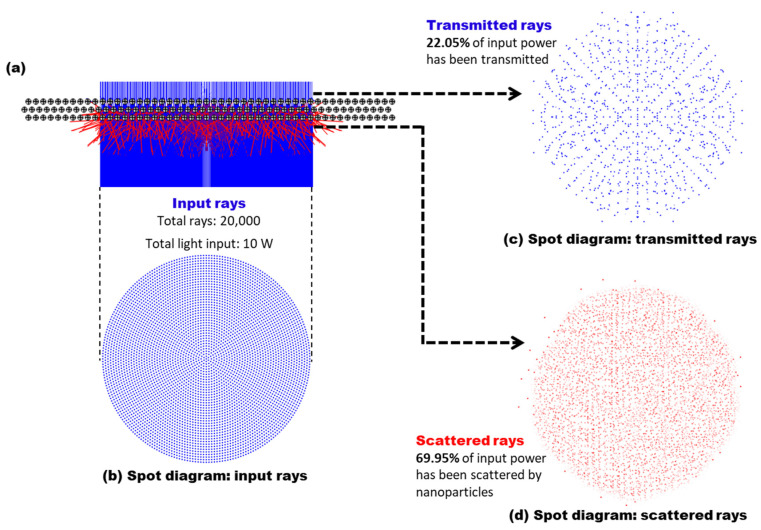
**Plasmonic light scattering by an array of metallic nanoparticles.** (**a**) A cross-sectional view shows three staggered layers of uniformly distributed conductive nanoparticles. Each nanoparticle has a diameter of 50 nm. Within each layer, the nanoparticles are uniformly distributed 20 nm apart from each other. The three layers are arranged in a staggered manner, separated by a vertical height of 25 nm. Spot diagrams of (**b**) the input rays modelled as a circular beam with a total of 20,000 input light rays projected from the bottom onto the nanoparticle arrays, (**c**) the transmitted rays emerged from the top of the nanoparticle arrays, and (**d**) the scattered rays emerged from the bottom of the nanoparticle arrays. The light rays are scattered at an angular spread upon hitting the nanoparticles. Only 22.05% of the input power emerged from the top layer as transmitted rays, while the scattered rays emerged from the bottom of the nanoparticle arrays at 69.95% of the input power. From the perspective of a FEOET device, the addition of a metallic nanoparticle layer above the low-quality TiOPc layer causes plasmonic light scattering to redirect additional rays onto the TiOPc layer, greatly enhancing its light absorption and photo-state conductivity.

To closely present plasmonic light scattering by the spin-coated nanoparticles experimentally fabricated, the simulation study assumed the nanoparticles to be arranged in three staggered arrays. A cross-sectional view of the nanoparticle arrays is shown in Figure 3a. Each nanoparticle (diameter of 50 nm) was uniformly distributed 20 nm apart from one another and the 50 × 50 arrays were stacked into three staggered layers, separated by a vertical spacing of 25 nm between each layer. Similarly, a circular light beam (diameter of 2 µm) was modelled to represent the input rays that passed through the photoconductive layer without light absorption. Figure 3b shows the spot diagram of the input light beam consisting of a total of 20,000 rays with a total input power of 10 W illuminated from the bottom onto the nanoparticle arrays. Part of the input rays passes through the staggered nanoparticle arrays while another part of the rays undergoes light scattering by the nanoparticles and redirects their optical pathways. Figure 3c,d present the spot diagrams of transmitted and scattered rays extracted on the surfaces right above and below the nanoparticle arrays, respectively. From the simulation results, the transmitted rays emerged out from the top of the nanoparticle arrays carrying with only 22.05% of the input power (Figure 3c). On the other hand, the scattered rays were detected to carry 69.95% of the input power upon hitting the arrayed nanoparticles and redirected their optical pathways onto the photoconductive layer beneath to additionally contribute to its photo-state conductivity (Figure 3d). It is also noted that the remaining 8% of the input power presents the power carried by the rays that keep propagating within the nanoparticle arrays as well as being lost due to the angular-dependent light absorption properties by the nanoparticles, as described in Figure 2.

From the perspective of a FEOET device, this simulation study shows that the addition of the metallic nanoparticles above the TiOPc layer gives rise to plasmonic light scattering over a wide angular spread and redirect the scattered rays onto the TiOPc layer with the increase of effective optical path lengths. Based on the simulation results in Figure 3, 69.95% of the input power can additionally contribute to increasing the light absorption onto the TiOPc layer and its photo-state conductivity, thus enhancing the optical DEP performance of the FEOET device. It is also worthwhile to note that the use of a denser or thicker layer of the nanoparticles would enable a significantly higher number of scattered rays, which can induce a much higher light absorption on the TiOPc layer, resulting in enhanced optical DEP performance (see Supplementary Material). This thickness-dependent study will be further discussed in Section 4.

### 3.2. Electric Field Distribution

Another simulation study was implemented to see the effect of the nanoparticle layer on electric field enhancement. The device was simply modelled to have an 8.0 µm thick TiOPc layer being coated with a 12.5 µm thick PDMS layer and an oil medium (height of 500 µm). A 100 V DC voltage was applied at both end planes, separated by a 1.0 mm gap, which creates a uniform electric field along a lateral direction. To represent a low-quality photoconductive property of the TiOPc layer, its conductivity at the photo and dark states was assumed as σ_photo_ = 1 × 10^−7^ S/m and σ_dark_ =1 × 10^−8^ S/m, only a 10-fold conductivity ratio, i.e., *b* = σ_photo_/σ_dark_ = 10.

**Figure 4:**
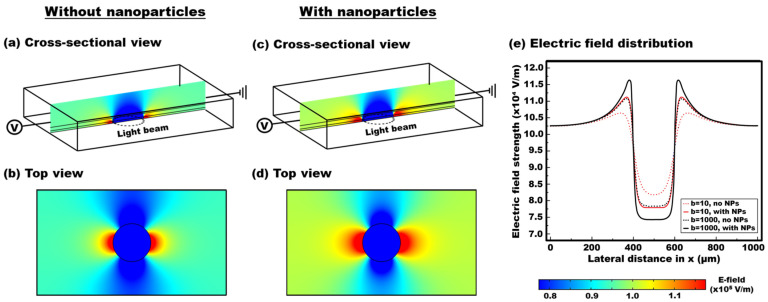
**Simulation results of electric field distribution to demonstrate plasmonic-enhanced DEP performance of FEOET devices**. (**a**–**d**) Cross-sectional and top views of the electric field strengths are shown under the illumination of a circular light beam for the device without and with plasmonic nanoparticles. The photoconductivity ratio is assumed at *b* = 10. By using the arrayed nanoparticle layers above the TiOPc layer, electric field strengths can be significantly enhanced at both sides of the light beam in the lateral direction, even at the same light illumination. (**e**) The field strength data are extracted on the surface of the dielectric layer. A stronger field gradient can be created with the presence of plasmonic nanoparticles. This simulation study was repeated for another conductivity ratio at *b* = 1000 to represent the use of high-quality photoconductive material (see black solid and dotted curves). By using plasmonic nanoparticles as light scattering elements, even low-quality photoconductive materials at *b* = 10 can induce the strong field gradient as comparable as high-quality photoconductive ones at *b* = 1000.

Figure 4a,b show both the cross-sectional and top views of the electric field strengths disturbed by a circular light beam (diameter of 200 µm) illuminated in the middle of the device without the presence of a nanoparticle layer. Another identical device was also modelled with the additional three staggered layers of the nanoparticles uniformly distributed 20 nm apart from each other above the TiOPc layer. Similarly, both the cross-sectional and top views of the field strengths for the device with the stacked nanoparticles are presented in Figure 4c and Figure 4d, respectively. For the FEOET device with the plasmonic nanoparticles, a much stronger electric field strength can be created near the edges of the circular light beam along a lateral direction, compared to the ones without nanoparticles. To clearly see the effect of plasmonic nanoparticles on electric field gradient, the field strengths were extracted along a lateral direction above the dielectric layer of the devices without and with the addition of the nanoparticles. The field distribution data are plotted in Figure 4e. It is clearly observed that the electric field gradient could be greatly enhanced near the edges of a circular light beam when plasmonic nanoparticles are present (see red dotted and solid curves for *b* = σ_photo_/σ_dark_ = 10). This enhanced field gradient penetrates to the oil layer and greatly disrupts the originally balanced electric field pattern around the droplet. As a result, a much larger dielectrophoretic force can be created for effective droplet actuation even under the same light illumination on FEOET devices.

This simulation study was also repeated for another conductivity ratio at *b* = 1000 to represent the use of amorphous silicon (a-Si) as a high-quality photoconductive material [37,40]. Similarly, the field gradient enhancement is obtained with the inclusion of plasmonic nanoparticles (see black dotted and solid curves of Figure 4e). One interesting observation from Figure 4e is that, by using plasmonic nanoparticles as light scattering elements, even low-quality photoconductive materials at *b* = 10 can induce the field gradient as comparable as the one by high-quality photoconductive materials at *b* = 1000, showing a dramatic increase in the field non-uniformity.

## 4. Experimental Study

To verify the numerical simulation results discussed in the previous sections, practical demonstrations of TiOPc light absorption performance and DEP force enhancements with the use of plasmonic nanoparticles are presented by measuring the spectrophotometric absorbance of the TiOPc as well as analyzing light-actuated droplet dynamics.

### 4.1. Spectrophotometric Absorbance of the TiOPc

**Figure 5:**
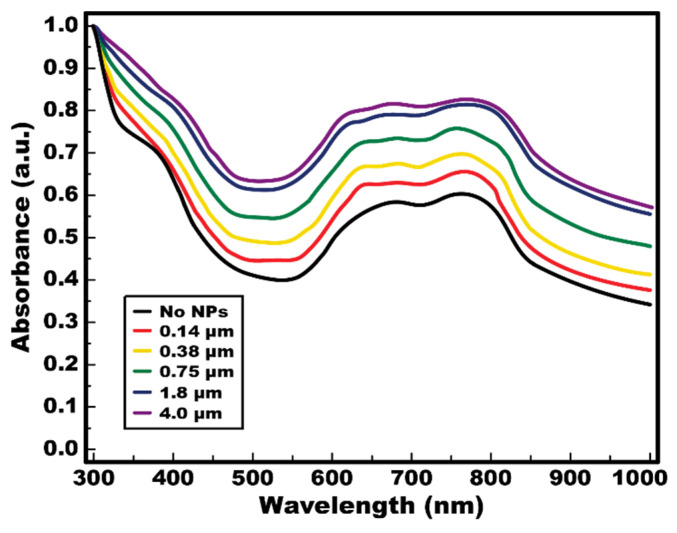
**Spectrophotometric absorbance measurement of a TiOPc layer.** The absorption spectra of an 8.0-µm TiOPc layer were measured to study the influence of various thicknesses of the nanoparticle layer on light absorption of the TiOPc layer. The nanoparticle layers were prepared from 0.14 µm to 4.0 µm by simply adjusting a spin-coating speed. From the measurements, light absorption is observed to be much larger with the presence of a nanoparticle layer as compared to a pure TiOPc layer (no NPs). Notably, light absorption is further enhanced with a thicker nanoparticle layer. However, the absorption spectra of TiOPc with 1.8-µm and 4.0-µm-thick nanoparticle layers are very close to each other, implying saturated scattering performance of a nanoparticle layer as increasing its thickness.

A spectrophotometer (Spark, Tecan Group Ltd., Zurich, Switzerland) was used to measure the absorption spectrum of the TiOPc layer used in experiments. To explore the thickness effect of the nanoparticle layer on the absorption spectrum of the TiOPc, the layer thickness was varied from 0.14 µm to 4.0 µm by adjusting a spin-coating speed. A black curve in Figure 5 presents the absorption spectrum of the pure TiOPc layer (i.e., no nanoparticles added), where a relatively higher light absorption can be seen within the wavelengths from 600 nm to 800 nm as similarly reported in previous studies [45,46]. With the presence of a nanoparticle layer, the light absorption onto the TiOPc layer is observed to be much larger as compared to the pure layer of TiOPc. Light absorption can also be further enlarged as the thickness of the nanoparticle layer increases. This observation is caused by the increase in plasmonic light scattering as more light rays were angularly scattered and redirected back onto the TiOPc layer by a larger number of nanoparticles presented with the increase in the layer thickness. Another interesting observation is that the absorption spectra for 1.8 µm (blue) and 4.0 µm (purple) thick nanoparticle layers are very close to each other, indicating the plasmonic scattering performance saturated with the thickness of the nanoparticle layer. More light rays are propagated within the increasingly thick and dense nanoparticle layer instead of being scattered back to the TiOPc layer. As such, the enlargement in light absorption onto the TiOPc starts to saturate even with the increase in nanoparticle layer thickness.

### 4.2. Light-Actuated Droplet Dynamics

For this experimental study, a 1.5-μL deionized water droplet was loaded onto a FEOET device filled with mineral oil. The nanoparticle layer was prepared with its thickness varied from 0.14 μm to 4.0 μm by adjusting a spin-coating speed to understand the thickness effect on plasmonic-enhanced DEP performance. A bias voltage was laterally applied across the device at 200 V/mm. At the same time, a circular laser beam was projected onto the TiOPc layer at a position adjacent to the droplet. The droplet’s movement from its stationary position to complete stop was recorded using a high-speed camera (Fastcam Mini AX200, Photron, Tokyo, Japan).

**Figure 6:**
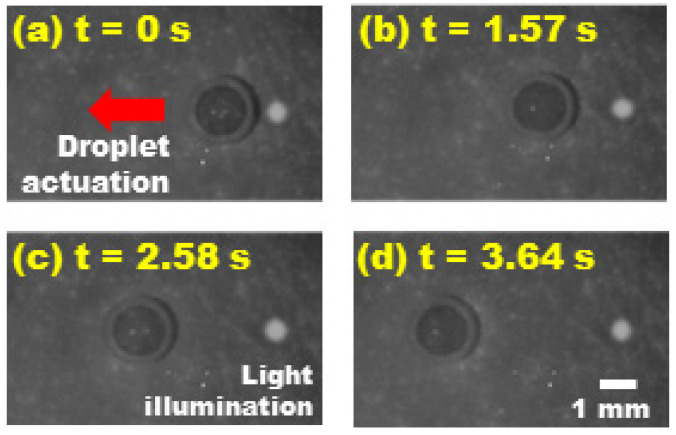
**Video snapshots of light-actuated DEP droplet movement on a FEOET device with a 4.0 µm nanoparticle layer.** (**a**) A laser beam (4.0 mW, 635 nm, 0.9 mm spot size) was illuminated near an edge of the droplet. (**b**) The droplet was repelled away from the light illumination area. (**c**) It reached its maximum instantaneous speed of 1.90 mm/s at *x* = 2.29 mm away from its original spot. (**d**) The droplet completely stopped at *x* = 3.44 mm.

**Figure 7:**
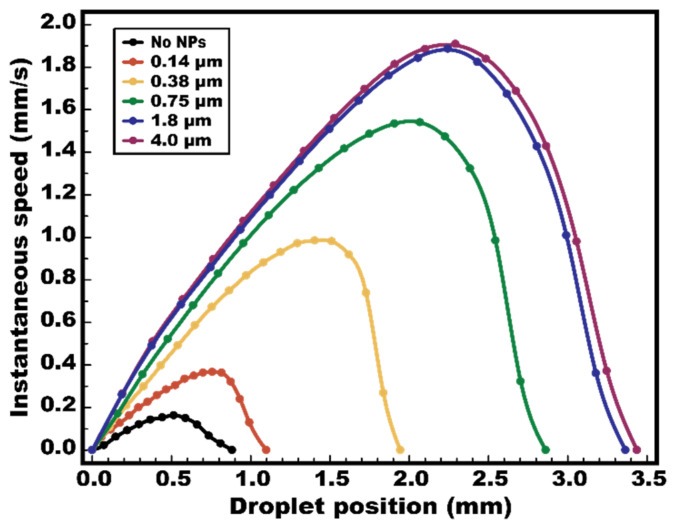
**Experimental results of optical DEP actuation.** FEOET devices were prepared with various thicknesses of a nanoparticle layer from 0 to 4.0 μm. The moving speeds of a 1.5 µL de-ionized water droplet were measured and recorded at its corresponding positions while a laser beam was illuminated near one edge of the droplet. With no nanoparticles, the droplet’s moving speed reached a peak of 0.163 mm/s. However, by using a 4.0 µm nanoparticle layer on a device, a maximum speed of 1.90 mm/s (>11-fold improvement) was achieved.

Figure 6 depicts video snapshots of the droplet being repelled away from a laser beam illumination nearby the droplet on the device with a 4.0 µm thick nanoparticle layer. With the use of plasmonic nanoparticles as light scattering elements, the droplet underwent its maximum speed of 1.90 mm/s at the position of 2.29 mm (Figure 6c), before coming to a complete stop at a location of 3.44 mm away from its origin (Figure 6d). For comparative study, this test was repeated with various thicknesses of the nanoparticle layer, while keeping the same experimental conditions (i.e., identical droplet size, electric field strength applied, light intensity). Figure 7 shows the instantaneous speed profiles of the droplet experimentally measured at its corresponding positions. For the device without nanoparticles, the droplet’s movement was just peaked at the position of 0.515 mm at a speed of as low as 0.163 mm/s before stopping at 0.883 mm away from its original spot. It is also interesting to note that the thicker the nanoparticle layer used, the higher the instantaneous actuation speed of a droplet can be achieved. This is because thicker nanoparticle layers enable more plasmonic light scattering and redirection onto the TiOPc layer to enhance its light absorption. As a result, a photo-state conductivity of the TiOPc layer largely increases, leading to improvement in the light-actuated DEP performance of the FEOET device. Additionally, it is interesting to observe a droplet speed saturation in Figure 7 as the nanoparticle layer thickness increases from 1.8 µm to 4.0 µm. This can be explained by the scattering performance saturated by the thickness of the nanoparticle layer, as supported in Figure 5. The increasingly thick nanoparticle layer resulted in more light rays being propagated within the layer instead of being scattered back to the photoconductive layer. Therefore, the enhancement in droplet speed begins to saturate even with the increase in nanoparticle layer thickness.

This light-actuated droplet dynamics study has experimentally validated the significant improvement in dielectrophoretic droplet manipulation by utilizing plasmonic nanoparticles. Even with a poor photoconductive material such as TiOPc, plasmonic light scattering induced by the nanoparticles has enabled a more than 11-fold increase in instantaneous actuation speed of a droplet than the one without any nanoparticles. In addition, this 11-fold speed improvement at 1.90 mm/s presents a similar comparison to the results reported in the previous FEOET studies for which a high-quality a-Si material was used [30,31], despite being achieved using a low-quality TiOPc layer.

## 5. Conclusions

A plasmonic-enhanced FEOET platform has been developed for effective DEP manipulation of oil-immersed aqueous droplets on a light-patterned photoconductive surface. The proposed use of the layer of metal nanoparticles on a photoconductive layer enables light to undergo wide-angle plasmonic light scattering. This results in an effective increase in their optical path lengths as a higher number of light rays can be absorbed by the photoconductive layer, leading to an enhanced optical DEP performance. Furthermore, the use of a low-quality TiOPc as a polymer-based photoconductive material in this study allows us to clearly understand the effects of plasmonic nanoparticles on enhancement in optical DEP droplet actuation performance.

3D numerical simulation studies were conducted to investigate how light rays are being scattered by nanoparticles and understand the plasmonic effects on improving DEP performance. The simulation data were verified by spectrophotometric measurements of light absorption enhanced by adding plasmonic nanoparticles on a poor-quality TiOPc layer and its thickness effect on light absorption. Furthermore, experimental studies of light-induced DEP droplet actuation also support the plasmonic-enhanced DEP performance. A maximum moving speed of the droplet at 1.90 mm/s can be attained with the use of the nanoparticles layer with a thickness of 4.0 µm in the FEOET device, which shows an 11-fold faster speed when compared to an identical device without the nanoparticle layer. Both simulation and experimental studies have demonstrated that the use of metallic nanoparticles as plasmonic light scattering elements can considerably enlarge optical DEP performance even with the use of a poor-quality TiOPc photoconductive material. This plasmonic-enhanced FEOET technology can considerably improve light-driven dielectrophoretic performance even by using low-quality photoconductive materials, thus providing low-cost, easy-fabrication solutions for various droplet-based microfluidic applications.

## Data Availability

The data presented in this study are available on request from the corresponding author.

## Supplementary Materials

The following are available online at https://www.mdpi.com/article/10.3390/mi13010112/s1, Figure S1: Plasmonic light scattering on a single layer of metallic nanoparticles, Figure S2: Plasmonic light scattering on two layers of metallic nanoparticles, Table S1: Table of comparison for simulations with different numbers of stacked nanoparticle arrays.
