# Supplementary material for: Optical Dielectrophoretic (DEP) Manipulation of Oil-Immersed Aqueous Droplets on a Plasmonic-Enhanced Photoconductive Surface
Source: Micromachines (Basel). 2022 Jan 11;13(1):112. doi: 10.3390/mi13010112 (PMC8777958; doi:10.3390/mi13010112)
Supplement: Supplementary file 1 [file micromachines-13-00112-s001.zip › micromachines-1543295-supplementary.pdf]

Supplementary material for

Optical dielectrophoretic (DEP) manipulation of oil-immersed aqueous droplets on a plasmonic-enhanced photoconductive surface

Si Kuan Thio and Sung-Yong Park*

Further simulation studies on plasmonic light scattering were conducted to understand how the nanoparticle thickness (i.e., more numbers of stacked nanoparticle arrays) affects the number of scattered rays. Therefore, the same simulation study (as discussed in Figure 3) was repeated twice by assuming (1) a single layer of nanoparticle array and (2) two staggered layers of nanoparticle arrays.

1. Plasmonic light scattering on a single layer of metallic nanoparticles

For the simulation study with a single layer of nanoparticle array, each nanoparticle was modelled as 50 nm in diameter, and uniformly distributed 20 nm apart from one another in a 50 × 50 array. Similarly, a circular light beam with 2 μm in diameter was set to represent the input rays that passed through the photoconductive layer without light absorption. A cross-sectional view of the nanoparticle

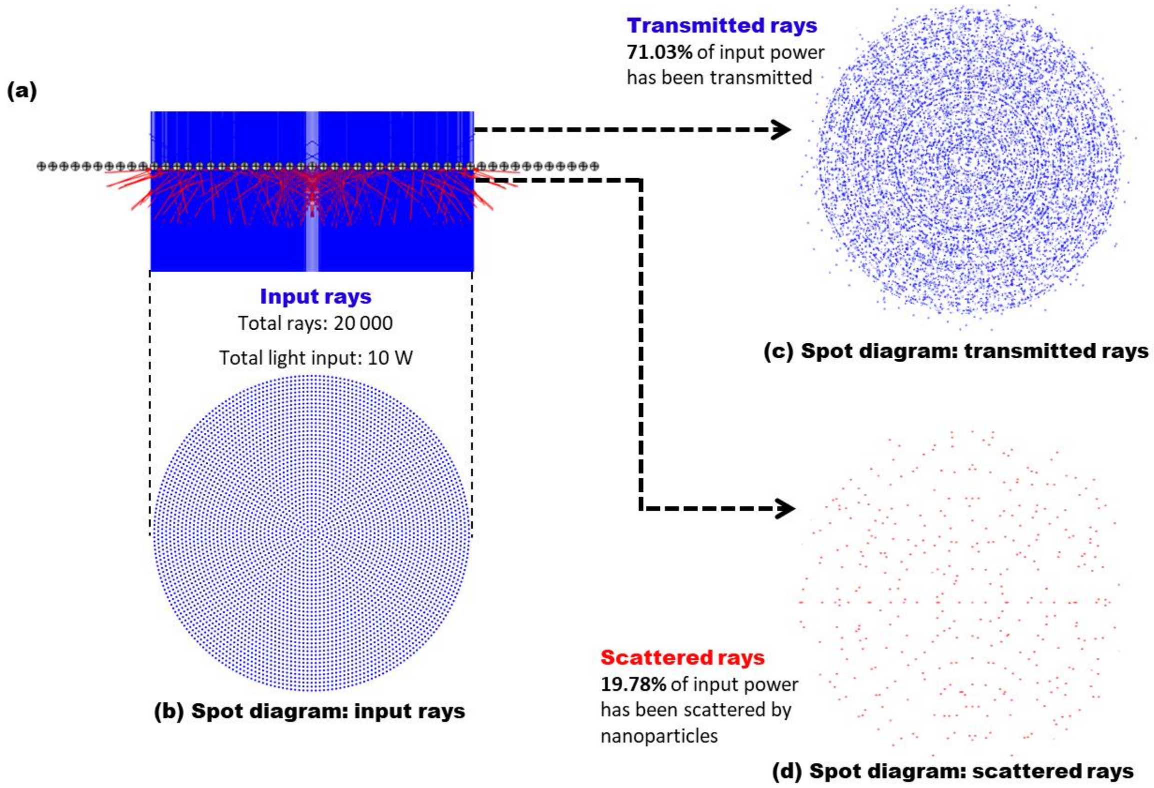

Figure S1. Plasmonic light scattering on a single layer of metallic nanoparticles. (a) A cross-sectional view shows a single layer of uniformly distributed conductive nanoparticles. Each nanoparticle has a diameter of 50 nm and uniformly distributed 20 nm apart from each other. Spot diagrams of (b) the input rays modelled as a circular beam with a total of 20 000 input light rays projected from the bottom onto the nanoparticle arrays, (c) the transmitted rays emerged from the top of the nanoparticle array, and (d) the scattered rays emerged from the bottom of the nanoparticle array. With a single layer of nanoparticles, only 19.78% of the input power emerged from the bottom of the nanoparticle array as scattered rays, while 71.03% of the input power emerged from the top as transmitted rays.

array is shown in Figure S1(a). The spot diagram of the input light beam shown in Figure S1(b) consists of a total of 20 000 rays with a total input power of 10 W illuminated from the bottom onto the nanoparticle array. Part of the input rays pass through the nanoparticle array as transmitted rays, while another part of the rays undergo light scattering by the nanoparticles and re-direct their optical pathways. Figure S1(c) and (d) present the spot diagrams of transmitted and scattered rays extracted on the surfaces right above and below the nanoparticle array, respectively. From this simulation results, the transmitted rays emerged out from the top of the array carrying with 71.03% of the input power (Figure S1c). On the other hand, the scattered rays were detected with 19.78% of the input power upon hitting the nanoparticles (Figure S1d). These percentages are considerably lower as compared to the simulation study (refer to Figure 3) with 3 staggered arrays of nanoparticles (22.05% for transmitted rays, 69.95% for scattered rays). This has proven that using more numbers of the stacked nanoparticle arrays would enable a significantly higher number of scattered rays.

2. Plasmonic light scattering on 2 layers of metallic nanoparticles

Similarly, the simulation study with 2 layers of nanoparticle arrays was conducted with the same simulation conditions, where each nanoparticle was modelled as 50 nm in diameter, and uniformly distributed 20 nm apart from one another. The two 50 × 50 arrays were stacked together with a vertical height of 25 nm between each layer. A circular light beam with 2 μm in diameter was modelled to represent the input rays that passed through the photoconductive layer without light absorption. Figure S2(a) represents the cross-sectional view of the nanoparticle arrays. Next, a circular light beam

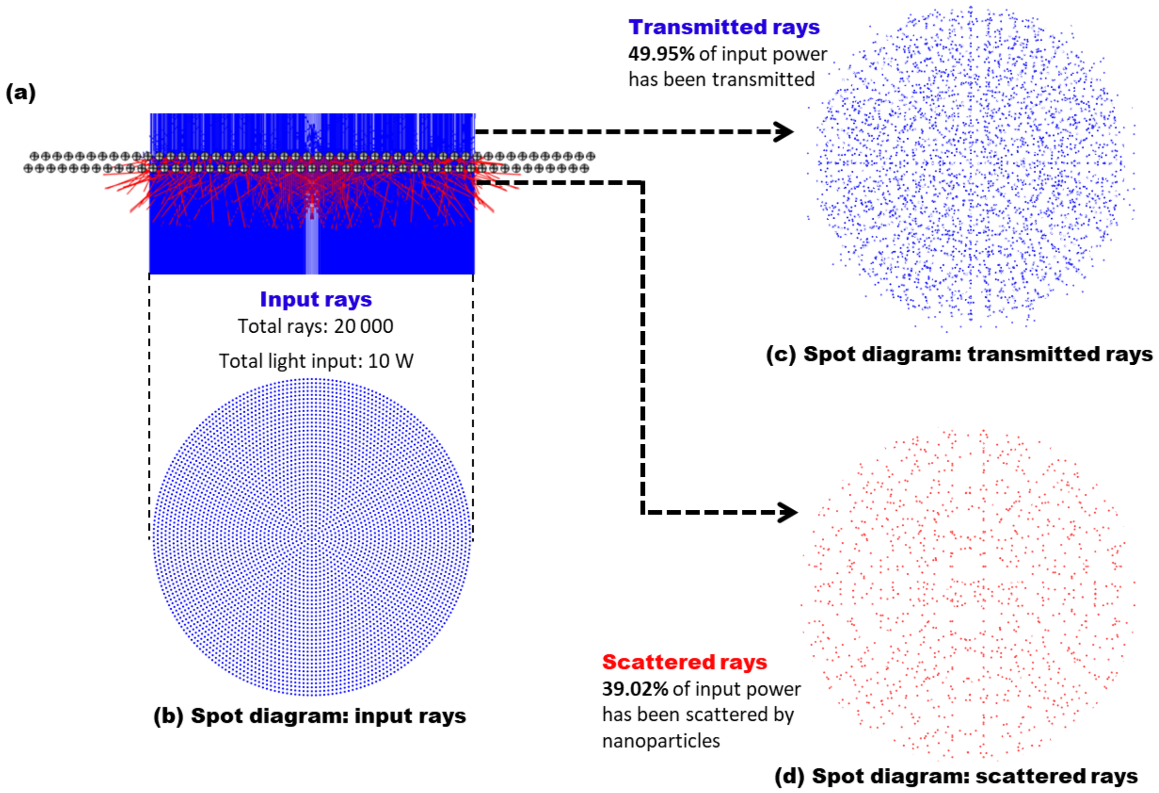

Figure S2. Plasmonic light scattering on 2 layers of metallic nanoparticles. (**a**) A cross-sectional view shows 2 staggered layers of uniformly distributed conductive nanoparticles. Each nanoparticle has a diameter of 50 nm and uniformly distributed 20 nm apart from each other. The 2 layers are separated by a vertical height of 25 nm. Spot diagrams of (**b**) the input rays modelled as a circular beam with a total of 20 000 input light rays projected from the bottom onto the nanoparticle arrays, (**c**) the transmitted rays emerged from the top of the nanoparticle arrays, and (**d**) the scattered rays emerged from the bottom of the nanoparticle arrays. With a single layer of nanoparticles, only 39.02% of the input power emerged from the bottom of the nanoparticle array as scattered rays, while 49.95% of the input power emerged from the top as transmitted rays.

(2 µm in diameter) was modelled as input rays that passed through the photoconductive layer without light absorption. The spot diagram of the input light beam shown in Figure S2(b) consists of a total of 20 000 rays with a total input power of 10 W illuminated from the bottom onto the nanoparticle arrays. The input rays that passed through the nanoparticle arrays as transmitted rays are shown in the spot diagram of Figure S2(c). Similarly, the rays undergo light scattering by the nanoparticles and re-direct their optical pathways are presented in the spot diagram of Figure S2(d). From this simulation results, the transmitted rays emerged out from the top of the array carrying with 49.95% of the input power (Figure S2c). Likewise, 39.02% of the input power were detected as scattered rays (Figure S2d). These percentages are higher when compared to the result in Figure S1 but lower in comparison to the simulation study (refer to Figure 3) with 3 staggered arrays of nanoparticles (22.05% for transmitted rays, 69.95% for scattered rays).

The above simulation studies (summarized in Table S1 for easier visualization) have further verified that the use of a denser or thicker layer (i.e., more numbers of the stacked arrays) of the nanoparticles would enable a significantly higher number of scattered rays. As such, a much higher light absorption on the photoconductive layer can be induced, resulting in enhanced optical DEP performance.

Table S1. Table of comparison for simulations with different numbers of stacked nanoparticle arrays.

No. of stacked arrays	Cross-sectional view	Spot diagram	
		Transmitted rays	Scattered rays
1	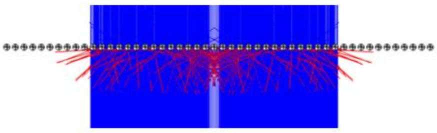	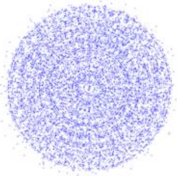 71.03% of input power	19.78% of input power
2	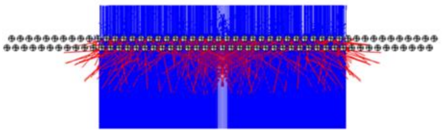	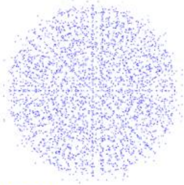 49.95% of input power	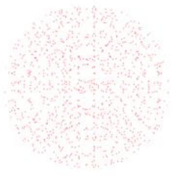 39.02% of input power
3	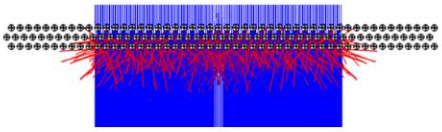	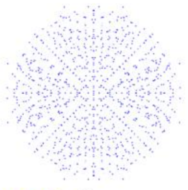 22.05% of input power	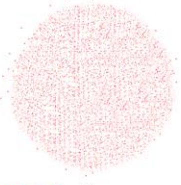 69.95% of input power
